# COVID-19 with high-sensitivity CRP associated with worse dynamic clinical parameters and outcomes

**DOI:** 10.3389/fmed.2024.1346646

**Published:** 2024-04-22

**Authors:** Kunapa Iam-Arunthai, Supat Chamnanchanunt, Pravinwan Thungthong, Anongnart Chinapha, Chajchawan Nakhahes, Tawatchai Suwanban, Tsukuru Umemura

**Affiliations:** ^1^Division of Hematology, Department of Medicine, Rajavithi Hospital, College of Medicine, Rangsit University, Bangkok, Thailand; ^2^Department of Clinical Tropical Medicine, Faculty of Tropical Medicine, Mahidol University, Bangkok, Thailand; ^3^Division of Infectious Diseases, Department of Medicine, Rajavithi Hospital, College of Medicine, Rangsit University, Bangkok, Thailand; ^4^Department of Medical Technology and Sciences, International University of Health and Welfare, Okawa, Japan

**Keywords:** COVID-19, Hs-CRP, mortality, clinical outcome, critical illness

## Abstract

**Objective:**

This study aimed to evaluate the relationship between high-sensitivity C-reactive protein (hsCRP) in hospitalized COVID-19 patients and their clinical outcomes, including trajectory of hsCRP changes during hospitalization.

**Method and results:**

Patients with positive COVID-19 tests between 2021 and 2023 were admitted to two hospitals. Among 184 adult patients, approximately half (47.3%) had elevated hsCRP levels upon admission, which defined as exceeding the laboratory-specific upper limit of test (> 5.0 mg/L). Clinical outcomes included critical illness, acute kidney injury, thrombotic events, intensive care unit (ICU) requirement, and death during hospitalization. Elevated hsCRP levels had a higher risk of ICU requirement than those with normal, 39.1% versus 16.5%; adjusted odds ratio (aOR), 2.3 [95% CI, 1.05–5.01]; *p* = 0.036. Patients with extremely high (≥2 times) hsCRP levels had aOR, 2.65 [95% CI, 1.09–6.45]; *p* < 0.001. On the fifth day hospitalization, patients with high hsCRP levels associated with acute kidney injury (aOR, 4.13 [95% CI, 1.30–13.08]; *p* = 0.016), ICU requirement (aOR, 2.67 [95%CI, 1.02–6.99]; *p* = 0.044), or death (aOR, 4.24 [95% CI, 1.38-12.99]; *p* = 0.011). The likelihood of worse clinical outcomes increased as hsCRP levels rose; patients with elevated hsCRP had lower overall survival rate than those with normal (*p* = 0.02). The subset of high hsCRP patients with high viral load also had a shorter half-life compared to those with normal hsCRP level (*p* = 0.003).

**Conclusion:**

Elevated hsCRP levels were found to be a significant predictor of ICU requirement, acute kidney injury, or death within 5 days after hospitalization in COVID-19 patients. This emphasized the importance of providing more intensive care management to patients with elevated hsCRP.

## Introduction

Coronavirus disease (COVID-19), identified first in December 2019, is caused by severe acute respiratory syndrome coronavirus 2 (SARS-CoV-2), an enveloped, positive-sense, single-stranded RNA virus belonging to the *Betacoronavirus* genus of the *Orthocoronavirinae* subfamily in the *Coronaviridae* family. Six types of coronaviruses (CoV-229, CoV-OC43, CoV-NL63, CoV-HKU1, SARS-CoV, and MERS-CoV) can cause human respiratory tract infection (1). The primary mode of transmission is person-to-person via direct contact and respiratory droplets. COVID-19 symptoms range from mild or undetectable symptoms to highly severe symptoms, including death (2). Disease severity and mortality rates are closely associated with comorbidities such as advanced age, diabetes, hypertension, and cardiovascular diseases (3). Several inflammation markers, including cytokines, nitrogen species, and mediators, have been documented to predict severity (4), and these markers widely use C-reactive protein (CRP) and high-sensitivity CRP (hsCRP) (5–7). Elevated levels of acute-phase proteins are associated with various cytokines, which indicate inflammation. Worse COVID-19 clinical outcomes are caused by cytokine storms and the formation of widespread microthrombi among multiple organ systems (8). Novel biomarkers are being investigated to predict clinical outcomes (9). The hsCRP test is widely used to assess cardiovascular disease risk or monitor inflammation in patients because hsCRP is more sensitive than CRP detection, which detects vessel inflammation. Theoretically, the COVID-19 virus can damage respiratory and vascular tissues. Several studies have demonstrated that elevated hsCRP levels are correlated with worse clinical outcomes in patients with COVID-19 infection, suggesting that hsCRP might be a valuable biomarker for predicting the severity of clinical outcomes (8, 10). However, the correlation between hsCRP and hospitalization is not fully understood. The dynamics of hsCRP levels in patients with COVID-19 infection are still being investigated. We hypothesized that hsCRP is associated with worse clinical outcomes among hospitalized COVID-19 patients. This study analyzed clinical data from two hospitals in Thailand to explore the association of hsCRP levels with all-cause mortality and the likelihood of hospital discharge.

## Methods

### Patient cohort and demographic data

This retrospective observational study was conducted at the Rajavithi and Rangsit (Rajavithi-2) Hospital. The study enrolled 184 adult patients in Thailand between 2021 and 2023. All patients had tested positive for SARS-CoV-2, confirmed by real-time polymerase chain reaction (RT-PCR) upon admission to the hospital. Pregnant women and patients who were chronically using anti-inflammatory drugs were excluded from the study. Demographic information was collected from the medical records of all adult patients (over 18 years old). Laboratory tests, including a complete blood count, blood chemistry panel (including renal and liver function tests), coagulation parameters, and measurements of acute phase reactants (including ferritin, ferritin, and D-dimer), were conducted. Radiological chest X-rays and computed tomography (CT) scans were performed. The Ethics Committee of Rajavithi Hospital approved this study (Number 198/2564).

### Molecular testing for SAR-CoV-2 by RT-PCR; Ct value method

Patients admitted with positive COVID-19 RT-PCR results were enrolled retrospectively. The Bio-Rad CFX96™ RT-PCR instrument constructs a real-time amplification curve based on the signal changes. The qualitative detection of the SARS-CoV-2 novel coronavirus at the nucleic acid level was reported in the FAM channel for ORF1ab of Ct value, N (labeled by VIC). A Ct value less than 25 was considered a low SARS-CoV-2 viral load (11, 12). High-sensitivity C-reactive protein (hsCRP) levels were measured using the Abbott Architect c16000 automated biochemical analyzer (Abbott Diagnostics, North Chicago, USA). The reference range for hsCRP was 0-5.0 mg/L. Adults with COVID-19 were categorized into two groups based on hsCRP levels at admission: those with values exceeding the referenced high cutoff of 5 mg/L were designated as the “high hsCRP group,” while all others were classified as the “low hsCRP group.” Measurements were tested on days 0, 3, 5, 7, 10, and 14.

### Clinical severity classifications

COVID-19 severity was classified as follows: moderate cases were considered to have clinical or radiological signs of lower respiratory disease and an oxygen saturation (SpO_2_) level of at least 94% on room air. Severe cases were defined as a SpO_2_ level of less than 94%, a respiratory rate of more than 30 breaths/min, or radiological findings of lung infiltrates of more than 50% of the chest X-ray. Critical cases included patients who presented with respiratory failure, septic shock, or multiple organ dysfunction. Complications included acute kidney injury and thrombosis events. Clinical outcomes were admission to the intensive care unit (ICU) or death during hospitalization.

### Statistical analysis

Categorical variables are presented as numbers and percentages with comparison by χ^2^ tests. Continuous variables were characterized by medians with interquartile ranges (IQRs) for non-normally distributed data. A Mann–Whitney *U*-test was performed for all non-normally distributed data. The longitudinal trajectory of the mean hsCRP level per day during hospitalization for all patients in each clinical outcome category is shown using the fitted values from the general linear model for each time point separately. Logistic regression models to estimate the odds of different clinical outcomes with the covariates in the multivariable models include age, sex, type 2 diabetes, dyspnea, and initial laboratory results for neutrophil–lymphocyte ratio (NLR), alanine transaminase (ALT), and estimated glomerular infiltration rate (eGFR). Kaplan–Meier curves were performed to estimate the overall survival rate. Statistical analysis was performed using SPSS program version 22.0 (Mahidol University license), with a significance level set at a *p*-value of ≤0.05.

**Figure 1:**
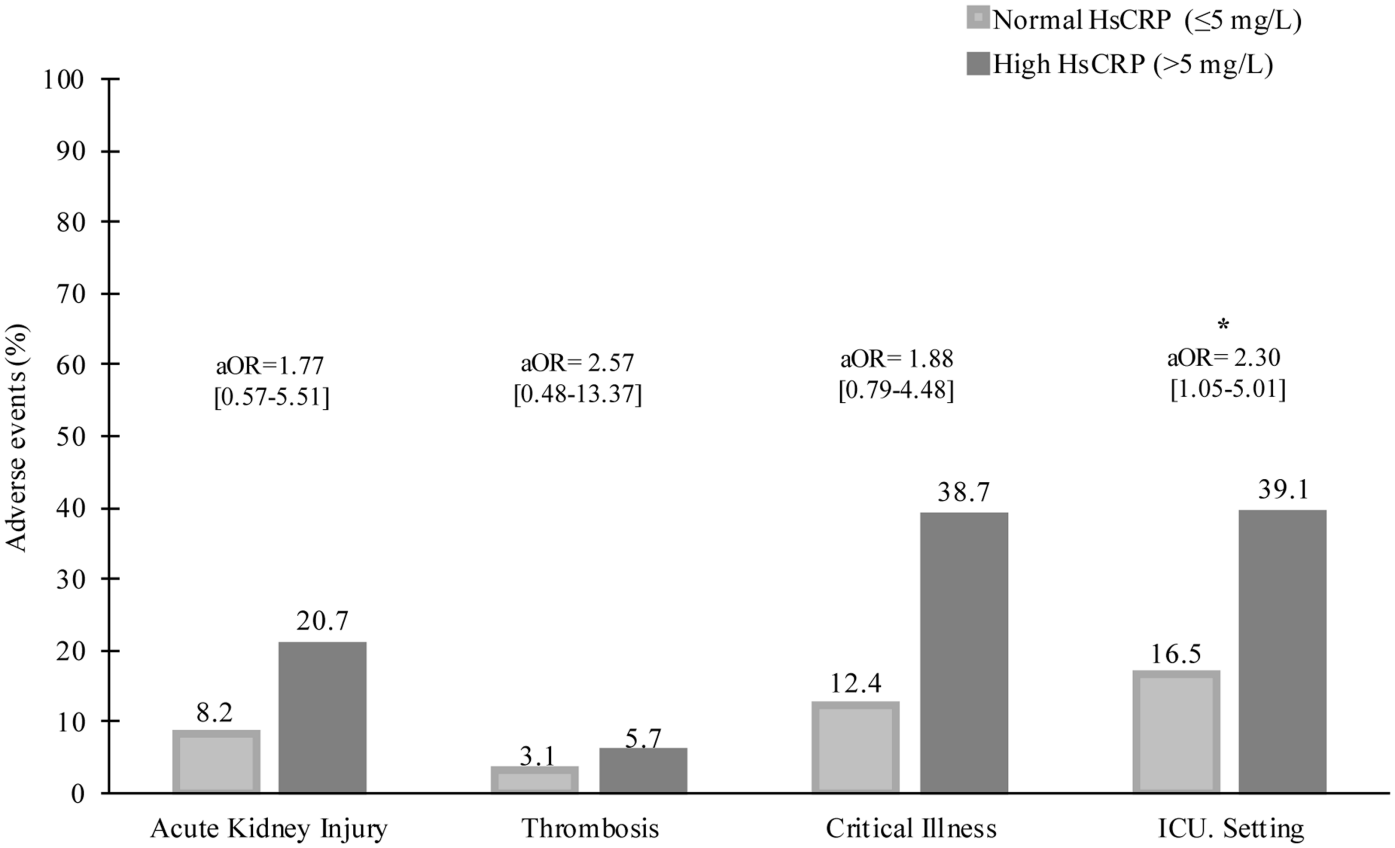
Baseline hsCRP levels and severe conditions during hospitalization. aOR, adjusted odds ratio, *p < 0.05.

## Results

Of the 184 hospitalized patients who tested positive for the SARS-CoV-2 virus between 2021 and 2023, 87 (47.3%) cases had high hsCRP levels (median = 11.20; [IQR = 7.03–18.31]). The median age of patients with high hsCRP was 61.0 [IQR = 52.0–72.0]. Patients with normal hsCRP were younger (median age, 55.0 [39.0–69.0] versus 61.0 [IQR = 51.0–72.0] years; *p* = 0.009) and had a lower incidence of type 2 diabetes (18.6% versus 34.5%, *p* = 0.014). The top four clinical presentations were fever (89.1%), cough (77.2%), dyspnea (67.4%), and sore throat (18.5%). Only dyspnea (*p* = 0.003) showed a statistically significant difference between patients with elevated and normal hsCRP levels at admission. Patients with elevated hsCRP level at admission had leukocytosis (7.27 [5.41–9.43] x 10^6^/L versus 6.24 [5.27–7.78] x 10^6^/L; *p* = 0.017), high absolute neutrophil count (ANC) (5,641 [4,011–8,307] cells/μL versus 4,349 [3,058–5,667] cell/μL), low absolute lymphocyte count (ALC) (1,128 [760–1,368] cells/μL versus 1,485 [942–1,960] cells/μL), high NLR (5.39 [3.49–8.47] versus 2.66 [1.71–4.86], elevated liver function test [ALT, AST], high D-dimer (1.22 [0.73–2.93] mg/L versus 0.66 [0.40–1.94] mg/L), high ferritin (1,657 [766–2,930] ng/dL versus 298 [128–954] ng/dL), high procalcitonin (0.2 [0.2–0.8] versus 0.1 [0–0.1], high LDH (513 [398–681] U/L versus 266 [213–357] U/L, low albumin (3.5 [3.2–3.7] g/dL versus 3.8 [3.5–4.1] g/dL), and low eGFR (72.0 [47.0–94.0] ml/min/1.73 m^2^ versus 95.0 [70.0–106.0] mL/min/1.73 m^2^) (*p* < 0.001 for each; Table 1).

**Table 1:** Baseline characteristics and clinical data of patients with COVID-19 stratified by HsCRP at admission.

Variables	All cases (*n*= 184)	High HsCRP (*n*= 87)	Low HsCRP (*n*= 97)	*p* value
Age (y)	59.0 (45.0−71.0)	61.0 (52.0−72.0)	55.0 (39.0−69.0)	0.009
Sex (female)	125 (67.9%)	52 (59.8.2%)	73 (75.3%)	0.025
BMI (Kg/m^2^)	26.5 (23.5−30.2)	26.7 (24.3−30.1)	26.2 (23.1−30.5)	0.412
Comorbidity, *n* (%)				
Hypertension	88 (47.8%)	45 (51.7%)	43 (44.3%)	0.316
Dyslipidemia	53 (28.8%)	30 (34.5%)	23 (23.7%)	0.107
Diabetes	48 (26.1%)	30 (34.5%)	18 (18.6%)	0.014
Chronic kidney diseases	13 (7.1%)	9 (10.3%)	4 (4.1%)	0.100
Cardiovascular diseases	6 (3.3%)	4 (4.6%)	2 (2.1%)	0.334
Hepatitis B infection	4 (2.2%)	2 (2.3%)	2 (2.1%)	0.912
Chronic pulmonary disease	3 (1.6%)	1 (1.1%)	2 (2.1%)	0.626
Cirrhosis	1 (0.5%)	0	1 (1.0%)	0.342
Presentation symptoms/signs				
Fever	164 (89.1%)	81 (93.1%)	83 (85.6%)	0.101
Cough	142 (77.2%)	65 (74.7%)	77 (79.4%)	0.451
Dyspnea	124 (67.4%)	68 (78.2%)	56 (57.7%)	0.003
Sore throat	34 (18.5%)	10 (11.5%)	24 (24.7%)	0.297
Runny nose	33 (17.9%)	11 (12.6%)	22 (22.7%)	0.076
Myalgia	22 (12.0%)	7 (8.0%)	15 (15.5%)	0.122
Diarrhea	23 (12.5%)	15 (17.2%)	8 (8.2%)	0.066
Anosmia	6 (3.3%)	2 (2.3%)	4 (4.1%)	0.487
Nausea/vomiting	5 (2.7%)	1 (1.1%)	4 (4.1%)	0.215
Laboratory data				
N2gene	21.9 (18.9−27.2)	22.4 (19.8−27.1)	21.5 (18.1−27.6)	0.246
Orf1Ab	23.8 (20.1−28.7)	25.2 (21.6−28.6)	22.6 (19.6−29.5)	0.094
Hemoglobin (g/dL)	12.7 (11.7−14.0)	12.6 (11.7−14.4)	12.8 (11.7−13.8)	0.635
WBC (x10^9^/L)	6.70 (5.38−8.37)	7.27 (5.41−9.43)	6.24 (5.27−7.78)	0.017
ANC (cells/μL)	4965 (3435−6342)	5641 (4011−8307)	4349 (3058−5667)	<0.001
ALC (cells/μL)	1206 (864−1679)	1128 (760−1368)	1485 (942−1960)	<0.001
NLR	3.96 (2.41−6.74)	5.39 (3.49−8.47)	2.66 (1.71−4.86)	<0.001
Platelet count (x10^9^/L)	218 (173−297)	221 (150−314)	213 (178−280)	0.830
PT (s) (*n*=98)	11.9 (11.4−12.8)	12.0 (11.5−12.8)	11.8 (11.3−12.8)	0.639
INR (*n*=98)	1.04 (1.00−1.13)	1.05 (1.01−1.13)	1.04 (0.99−1.12)	0.495
aPTT (s) (*n*=98)	26.5 (24.0−29.2)	26.3 (24.4−29.1)	26.5 (23.1−30.7)	0.767
ALT (U/L)	25.0 (17.0−48.0)	34.0 (22.0−65.0)	20.0 (13.0−49.0)	<0.001
AST (U/L)	36.0 (24.0−64.0)	56.0 (33.0−87.0)	27.0 (21.0−39.0)	<0.001
Albumin (g/dL)	3.6 (3.3−4.0)	3.5 (3.2−3.7)	3.8 (3.5−4.1)	<0.001
eGFR (ml/min/1.73m^2^)	86.5 (56.0−101.0)	72.0 (47.0−94.0)	95.0 (70.0−106.0)	<0.001
D-dimer (mg/L) (*n*=181)	0.95 (0.45−2.33)	1.22 (0.73−2.93)	0.66 (0.40−1.94)	<0.001
Ferritin (ng/dL) (*n*=154)	797 (267−2013)	1657 (766−2930)	298 (128−954)	<0.001
Procalcitonin (*n*=152)	0.4 (0.1−0.4)	0.2 (0.2−0.8)	0.1 (0.0−0.1)	<0.001
LDH (U/L) (*n*=182)	383 (253−563)	513 (398−681)	266 (213−357)	<0.001
Lactate (*n*=152)	1.50 (1.20−2.00)	1.70 (1.40−2.00)	1.40 (1.00−1.80)	0.004
Treatment				
Steriod	170 (92.4%)	83 (95.4%)	87 (89.7%)	0.0145
Oseltamivir	17 (9.2%)	9 (10.3%)	8 (8.2%)	0.624
lopinavir/ritonavir	120 (65.2%)	61 (70.1%)	59 (60.8%)	0.187
Ivermectin	43 (23.4%)	28 (32.2%)	15 (15.5%)	0.007

Data are number (percentage); ANC, Absolute neutrophil count; ALC, Absolute lymphocyte count; PT, Prothrombin time; aPTT, Activated prothrombin time.

### Clinical outcomes

**Figure 2:**
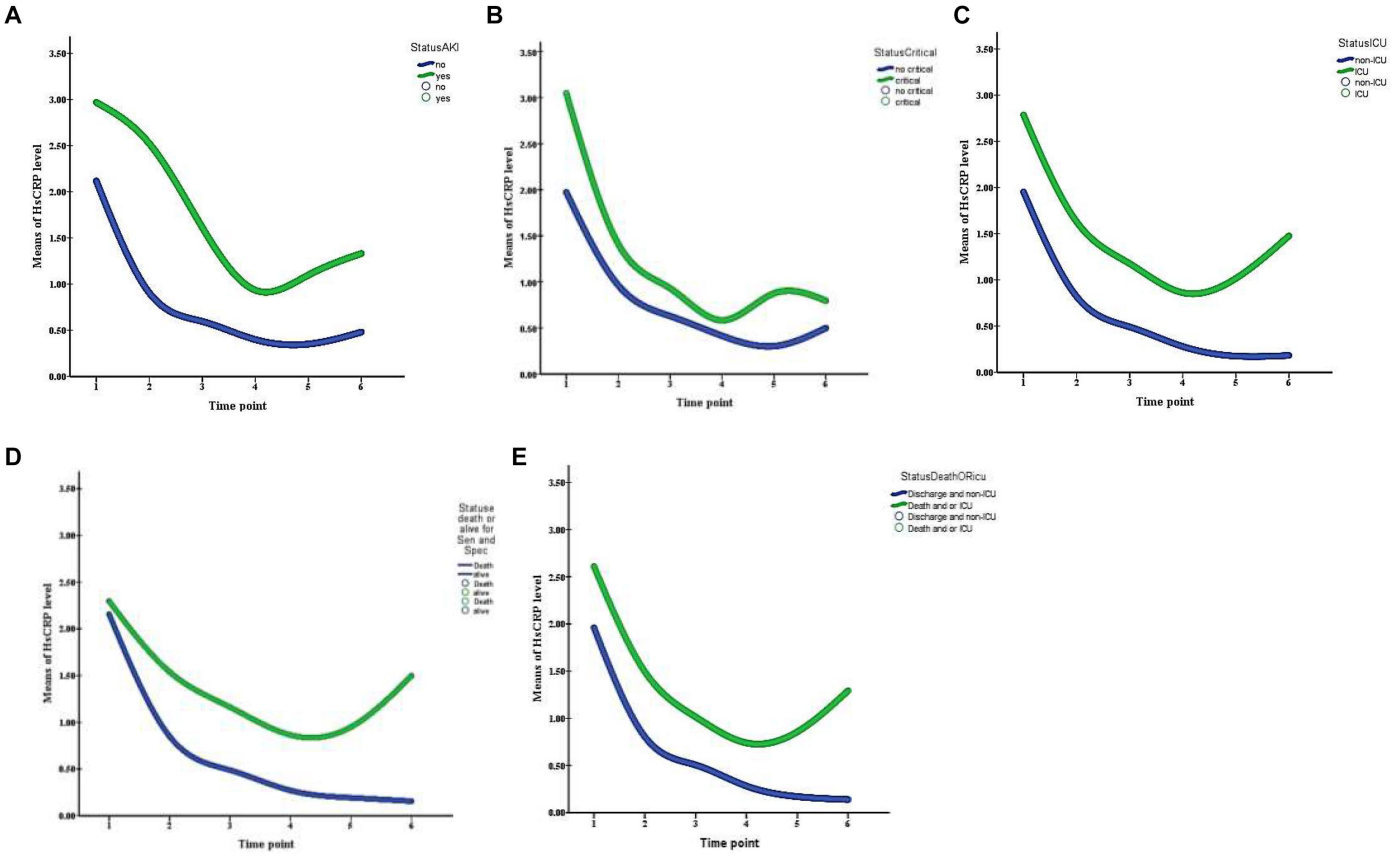
Trajectory of HsCRP levels during the first 14 day of hospitalization patients with COVID-19 infection are stratified by panel **(A)** acute kidney injury, **(B)** critical illness, **(C)** ICU setting, **(D)** death, and **(E)** death or ICU setting. Time points are identified as 1 (day 0), 2 (day 3), 3 (day 5), 4 (day 7), 5 (day 10), and 6 (day 14).

**Figure 3:**
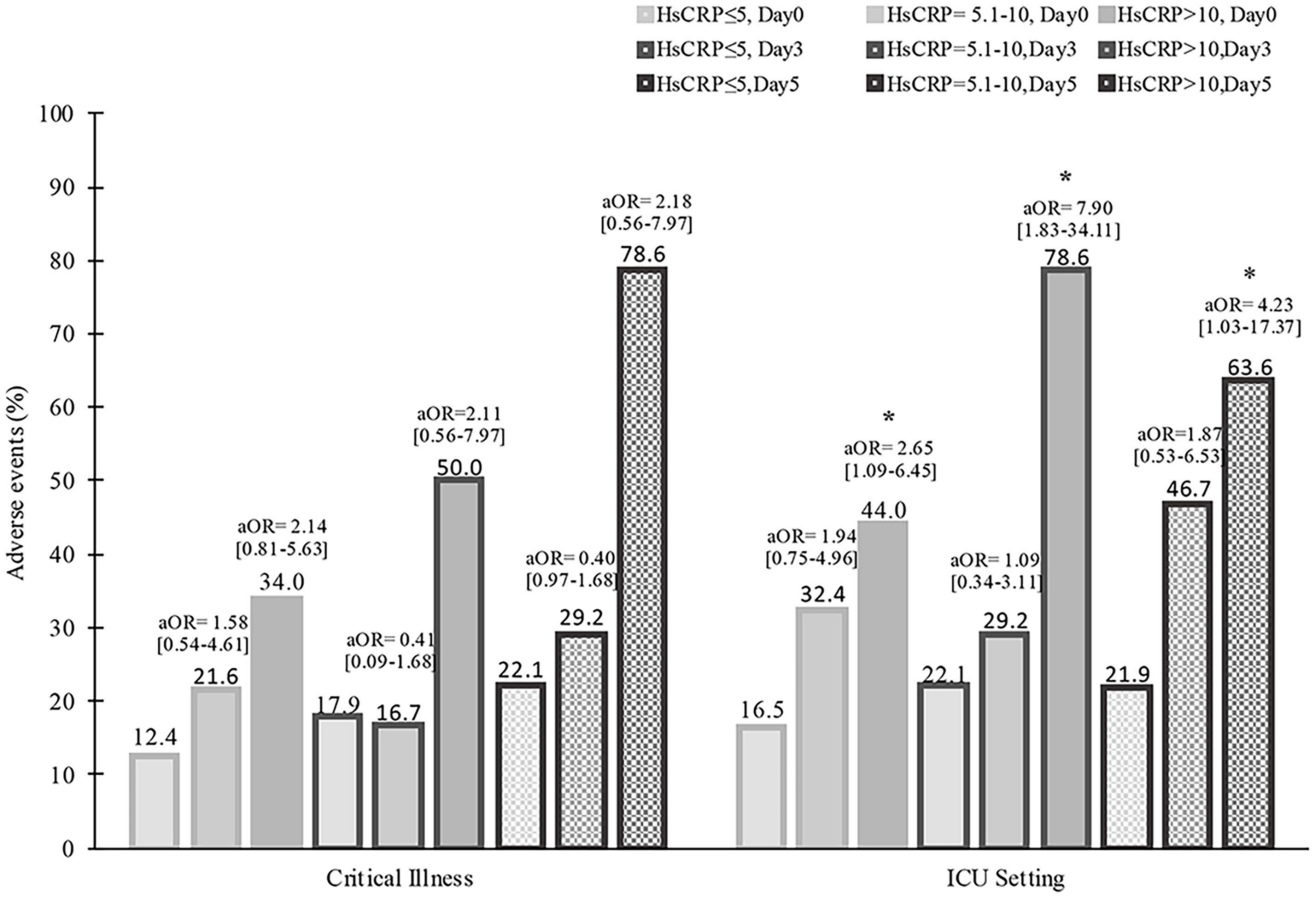
Serial high hsCRP curves to assess survival among patients with COVID-19 during 28-day hospitalization. aOR, adjusted odds ratio, *p < 0.05<.

During hospitalization, 37 (20.1%) patients had critical severity, 50 (27.2%) required ICU, 8 (4.3%) patients had a thrombotic event, and 26 (14.1%) developed acute kidney injury. Compared to those with normal hsCRP, individuals with elevated hsCRP were more likely to develop critical severity (38.7% versus 12.4%, *p* = 0.006), more often required ICU setting (39.1% versus 16.5%, *p* = 0.001), and had acute kidney injury (20.7% versus 8.2%, *p* = 0.016). Thrombotic events were not statistically different between elevated and normal hsCRP (5.7% versus 3.1%, *p* = 0.378). After adjusting the odd ratio for demographic, clinical presentation, and baseline laboratory values, elevated hsCRP levels at admission were associated with high adjusted odds of patients’ required ICU setting (aOR 2.30 [95% CI, 1.05–5.01]; *p* = 0.036) (Figure 1). During hospitalization, elevated hsCRP levels on the fifth day after hospitalization were associated with high adjusted odds of acute kidney injury (aOR 4.13 [95% CI, 1.30–13.08]; *p* = 0.016), required ICU setting (aOR 2.67 [95% CI, 1.02–6.99]; *p* = 0.044). The rest of the clinical outcomes (acute kidney injury, critical illness) showed significant only unadjusted odds (Table 2). All adjusted models had statistically significant differences (*p* < 0.001). The hsCRP level trajectory by acute kidney injury required ICU setting, and critical illness is presented in Figures 2A–C. Trend hsCRP levels generally dropped after the fifth day of hospitalization (time point number 3) (Figures 2D,E). Among 44% of patients with an hsCRP >10 mg/L, they had a high risk of requiring an ICU setting at admission (aOR 2.65 [95% CI, 1.09–6.45]; *p* < 0.001). The highest risk of required ICU setting was the third day of hospitalization among patients with very high hsCRP (aOR 7.90 [95% CI, 1.83–34.11]; *p* < 0.005), and the second aOR value was on the fifth day of hospitalization (aOR 4.23 [1.03–17.37]; *p* < 0.005) (Figure 3).

**Table 2:** Different HsCRP categories with unadjusted and adjusted OR for predicting patient outcome.

	Unadjusted OR (95% CI)	*p* value	Adjusted OR* (95% CI)	*p* value
Acute kidney injury** vs high HsCRP level follow-up period				
Low HsCRP	1.0		1.0	
Day 0	2.90 (1.19−7.06)	0.019	1.77 (0.57−5.51)	0.321
Day 3	5.76 (2.35−14.08)	<0.001	3.01 (0.95−9.54)	0.061
Day 5	7.67 (2.81−20.87)	<0.001	4.13 (1.30−13.08)	0.016
Critical illness** vs high HsCRP level follow-up period				
Low HsCRP	1.0		1.0	
Day 0	2.85 (1.33−6.12)	0.007	1.88 (0.79−4.48)	0.151
Day 3	1.86 (0.82−4.23)	0.136	0.88 (0.32−2.43)	0.813
Day 5	3.78 (1.52−9.10)	0.004	2.57 (0.91−7.22)	0.073
Thrombosis** vs high HsCRP level follow-up period				
Low HsCRP	1.0		1.0	
Day 0	1.91 (0.44−8.24)	0.385	2.57 (0.48−13.73)	0.268
Day 3	1.28 (0.24−6.64)	0.763	1.35 (0.22−8.25)	0.742
Day 5	0.93 (0.11−8.08)	0.950	1.53 (0.14−16.26)	0.722
ICU setting** vs high HsCRP level follow-up period				
Low HsCRP	1.0		1.0	
Day 0	3.24 (1.63−6.46)	0.001	2.30 (1.05−5.01)	0.036
Day 3	3.17 (1.50−6.71)	0.002	2.16 (0.89−4.96)	0.086
Day 5	4.15 (1.75−9.87)	0.001	2.67 (1.02−6.99)	0.044
Death** vs high HsCRP level follow-up period				
Low HsCRP	1.0		1.0	
Day 0	2.45 (1.31−4.55)	0.005	0.94 (0.40−2.18)	0.889
Day 3	2.83 (1.36−5.88)	0.005	1.13 (0.41−3.07)	0.804
Day 5	7.47 (2.91−19.08)	<0.001	4.24 (1.38−12.99)	0.011
Death or ICU setting** vs high HsCRP level follow-up period				
Low HsCRP	1.0		1.0	
Day 0	3.06 (1.65−5.67)	0.001	1.45 (0.66−3.18)	0.353
Day 3	3.01 (1.44−6.27)	0.003	1.39 (0.53−3.60)	0.498
Day 5	5.90 (2.31−15.01)	<0.001	3.26 (1.10−9.65)	0.033

*Odd ratios adjusted for age, sex, type 2 diabetes, dyspnea, neutrophil-lymphocyte ratio, ALT, eGFR. **The events of acute kidney injury, *n*= 26; critical illness, *n*= 37; thrombosis, *n*= 8; require ICU setting, *n*= 50; death, *n*= 65; death or require ICU setting, *n*= 72.

### HsCRP and all-cause mortality

**Figure 4:**
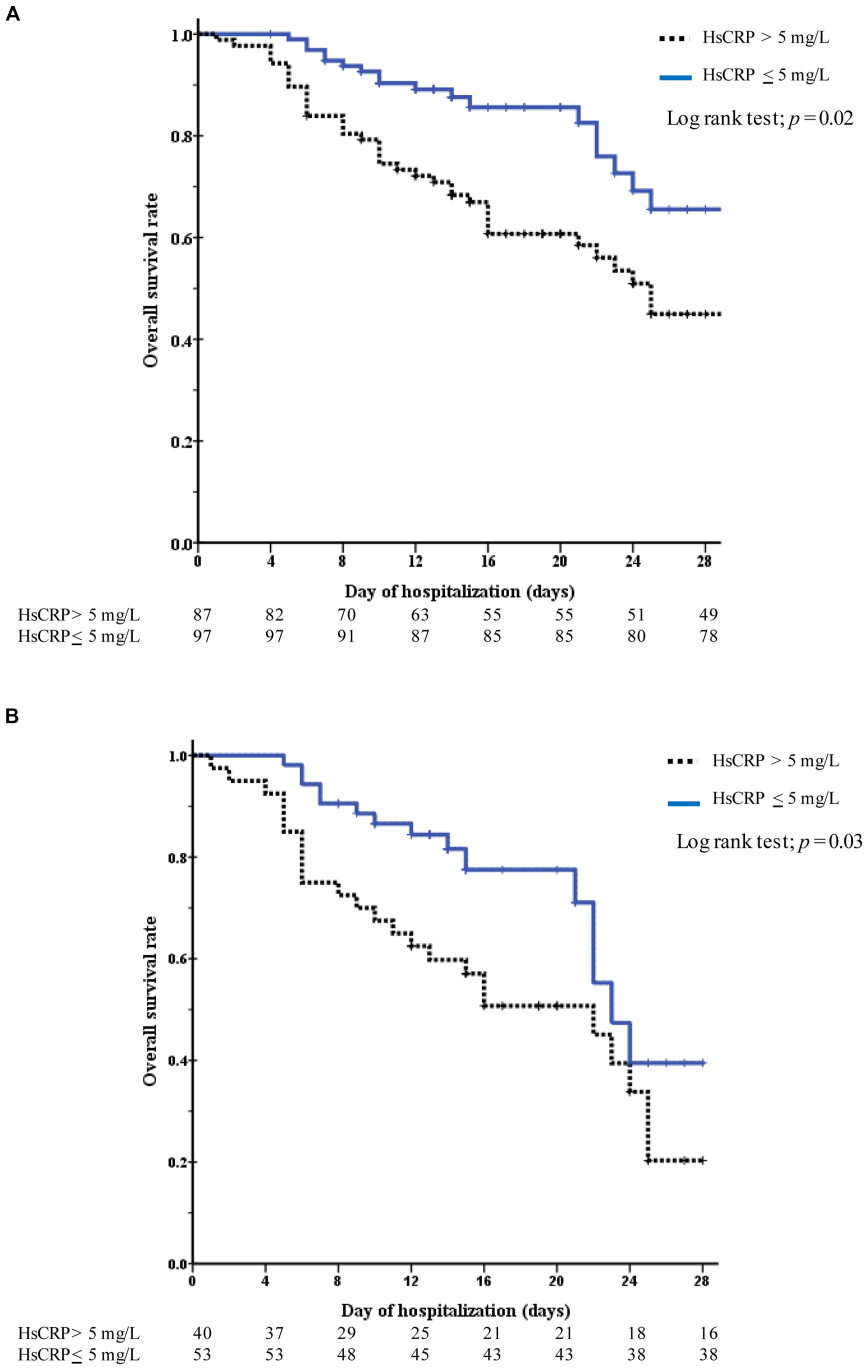
Kaplan–Meier curves to assess survival among patients with COVID-19 during 28-day hospitalization. The curves are color-coded stratifying to hsCRP levels (elevated hsCRP-blue blonde line, normal hsCRP-black dotted line). **(A)** Represents all patients and **(B)** represents only high COVID-19 viral test (the Ct value of <25).

Among the 184 hospitalized patients with COVID-19, 65 (35.3%) died, and 119 (64.7%) were discharged. Adjusted mortality was higher among patients with versus without elevated hsCRP levels on day 5 after hospitalization (aOR 4.24 [95% CI, 1.38–12.99]; *p* = 0.011). The multivariate-adjusted odds ratio of complications (death or required ICU setting) in patients with elevated hsCRP was higher than in those without on the fifth hospitalization (aOR 3.26 [95% CI, 1.10–9.65]; *p* = 0.033) (Table 2). Among 87 (47.3%) patients with elevated hsCRP levels at admission, the Kaplan–Meier survival curve demonstrated a significant difference between patients with elevated hsCRP and those without (log-rank test; *p* = 0.02). Similar to the subgroup analysis of patients with high viral load (Ct value <25) and elevated hsCRP, 93 (50.5%) high viral load patients had elevated hsCRP. The Kaplan–Meier curve showed a significantly lower overall survival rate in patients with elevated hsCRP than in those without (*p* = 0.003) (Figure 4).

## Discussion

Adult hospitalized with COVID-19 infection at two hospitals in Bangkok, Thailand, 47.3% had an elevated hsCRP level. Those patients with initially elevated hsCRP showed a 2-fold increased risk of ICU admission requirement. Interestingly, of serial hsCRP during hospitalization, those who tested for hsCRP on day 5 were associated with a 4-fold increased risk of acute kidney injury events. To the best of our knowledge, this study is the first to investigate the impact of dynamic hsCRP among patients with COVID-19 infection. Serial follow-up hsCRP levels within 5 days after hospitalization also showed an independent association with the increased requirement of an intensive hospital setting as well as death. The requirement for intensive care and mortality exhibited a dose-dependent correlation with the third hsCRP measurement in these patients. This finding is similar to the study of CRP concentration to show the association between respiratory failure and cardiovascular dysfunctions (13, 14). The patients were followed and categorized two times high hsCRP levels; those patients also showed between 4- and 7-fold increased ICU setting requirements. Patients with high hsCRP had a 3- to 4-fold increased risk of mortality (7). Our findings might be used to triage patients for immediate treatment and tailor treatment plans to individual patient needs. Interestingly, patients with high hsCRP levels had shorter survival rates, especially those with a high viral load. The present study suggests the association between patients with either high hsCRP or SAR-CoV-2 viral loads and high mortality due to high viral burden and high inflammation conditions (15–18). Therefore, the hsCRP level might guide the physician in estimating a prognosis and identifying poor prognosis patients with COVID-19 infection, especially new variants. The top four clinical presentations were fever, cough, dyspnea, and sore throat. The study by Lacobucci G et al. also showed that the omicron variant of COVID-19 had four common symptoms at admission (19). This epidemic data correlated with demographic data on the spread of COVID-19 in Thailand during 2021–2022 (20). As the survey results showed, the omicron variant of COVID-19 was a major species during this period. Thus, this omicron variant of COVID-19 affects patients with mild to moderate severity (21). Hospitalized patients might be undelegated admissions because of the decreasing severity of the disease. Tacoo et al. demonstrated that the number of required hospital beds declined during this situation (22). Similar to our study, 20% of hospitalized patients had severe forms, and 27% of patients occupied an ICU setting. Except for patients with elevated hsCRP levels, they were predominately transferred from the ward to the ICU. Patients with elevated hsCRP levels may need close monitoring. This study also showed that serial follow-up hsCRP is beneficial in patients with persistently elevated hsCRP levels within 5 days of hospitalization. Independently related to poor outcomes, hsCRP can persist within 72 h if the COVID-19 virus is damaged and related to a cytokine storm. Similar to Widasari N et al. they demonstrated that high hsCRP and neutrophil-to-lymphocyte ratio (NLR) correlated with high post-COVID-19 syndrome (23). NLR, neutrophil, and lymphocyte have been established markers significantly discriminating against COVID-19 patients with different progression and survival outcomes. This study adds to this evidence by demonstrating that hsCRP levels at admission possess similar discriminatory ability, suggesting its potential utility as a readily available and cost-effective marker. Most studies that used CRP demonstrated that this test is related to increased mortality rates due to the viral destruction of the vascular injury process. HsCRP is one of the most popular tests related to vascular complications. Our patient also had high acute kidney injury within 5 days after admission, which was explained by vascular injury events (14). However, small thrombotic events have been documented, especially in patients with preexisting diabetes and liver disease (24, 25). This is a limitation of the COVID-19 patient’s autopsy investigation because it is a highly contagious disease.

The limitation of this study was that it was a retrospective study, and the hsCRP test was not routinely and continuously performed in all patients. Some patients were excluded during data collection. Second, cardiovascular events depend on the diagnosis conducted by the attending physicians. However, our data were validated by two independent physicians. The bias or discordance is unknown. Third, no autopsy was performed because of the easy spread of viral properties. This may miss some data on death causes. Finally, we did not assess subsequent cardiovascular injury events among discharge patients.

## Conclusion

This study adds to the growing evidence that elevated hsCRP levels measured at admission significantly differentiate COVID-19 patients with poor clinical outcomes, such as needing ICU care or dying. Additionally, rising hsCRP levels within 5 days of hospitalization independently predicted these poor outcomes, even when accounting for vascular injury events like acute kidney injury. Notably, patients with high hsCRP and exceptionally high viral loads had shorter life expectancies. These findings support the potential of using hsCRP as an additional biomarker to represent a direct correlation with the severity of COVID-19 infection and predict patient outcomes.

## Data availability statement

The original contributions presented in the study are included in the article/supplementary material, further inquiries can be directed to the corresponding author.

## Ethics statement

The studies involving humans were approved by the Ethics Committee of Rajavithi Hospital. The studies were conducted in accordance with the local legislation and institutional requirements. The ethics committee/institutional review board waived the requirement of written informed consent for participation from the participants or the participants’ legal guardians/next of kin because this is a retrospective study.

## Author contributions

KI-a: Conceptualization, Data curation, Formal analysis, Funding acquisition, Investigation, Methodology, Project administration, Resources, Validation, Visualization, Writing – original draft, Writing – review & editing. SC: Conceptualization, Data curation, Formal analysis, Funding acquisition, Methodology, Project administration, Resources, Software, Supervision, Validation, Visualization, Writing – original draft, Writing – review & editing. PT: Conceptualization, Data curation, Investigation, Project administration, Validation, Visualization, Writing – review & editing. AC: Data curation, Investigation, Methodology, Project administration, Validation, Visualization, Writing – review & editing. CN: Investigation, Methodology, Validation, Visualization, Writing – review & editing. TS: Investigation, Methodology, Project administration, Validation, Visualization, Writing – review & editing. TU: Conceptualization, Data curation, Investigation, Project administration, Supervision, Validation, Visualization, Writing – review & editing.
